# Reply to the Letter to the Editor: Improving generative-AI performance in radiology through test-time compute

**DOI:** 10.1007/s00330-025-11665-3

**Published:** 2025-05-06

**Authors:** Sebastian Nowak, Babak Salam, Julian A. Luetkens, Alexander Isaak

**Affiliations:** 1 Department of Diagnostic and Interventional Radiology, University Hospital Bonn, Bonn, Germany; 2 Quantitative Imaging Lab Bonn (QILaB), University Hospital Bonn, Bonn, Germany

We thank Sorin et al for this valuable opinion letter and the opportunity for discussion.

In recent years, the progress of large language models (LLM) has been driven by the growing size of training data and compute used during the initial pre-training phase. Here, LLMs gain their vast knowledge from the enormous amounts of digital text available on the internet. However, as Sorin et al pointed out, research in LLMs has shifted from scaling training corpora and model sizes to optimizing inference. This shift emerged from concerns about the limitations of LLM progress, as the resources of digital text from the internet have become increasingly exhausted. Some proposed solutions, such as having models generate new training data, have proven less effective than anticipated and have raised concerns about the quality of internet text for training future frontier models, as the web becomes increasingly populated with content generated by earlier model generations [1].

It seems intuitive that thinking deeply about a problem before answering should lead to a better solution. As highlighted by Sorin et al, the traditional drivers of LLM progress can be augmented by compute used for reasoning tokens during inference—the process in which the trained model generates answers [2–4].

There are several approaches to training these specialized reasoning models. As an example, the Stanford-led study discussed by Sorin et al distilled reasoning skills from Google’s Gemini Flash Thinking into an open model from Alibaba by supervised fine-tuning on publicly available mathematics datasets, incorporating solutions and reasoning trails from Gemini [4]. Also, they introduced a guided decoding technique termed budget forcing, where the length of the model’s answer is controlled by suppressing the end token and replacing it with phrases like “Wait…”. This encourages the model to generate a longer, more elaborate response, potentially leading it to re-evaluate its initial output.

We agree that these techniques hold promise for narrowing the performance gap between open-source and closed-source models in the context of processing radiological reports and warrant further investigation. One might hypothesize that more complex tasks for an LLM, such as detecting errors and discrepancies in reports or evaluating descriptions of central venous catheter projection as correct or misplaced [5, 6], would benefit more from reasoning than simpler tasks, like text simplification or correcting basic spelling errors [7].

Importantly, unlike approaches based on reinforcement learning, budget forcing does not involve any additional post-training beyond the initial supervised model distillation. As Sorin et al highlighted, this is a method focused purely on test-time computation. Therefore, one might speculate that approaches like budget forcing can help extract correct answers based on pre-training knowledge more consistently. However, they may not introduce new knowledge and reasoning skills based on specialized radiological data.

Sorin et al highlight the recent and prominent example of DeepSeek-R1. Not only did DeepSeek-R1 rival OpenAI’s O1, but it also openly shared its model weights, parts of the optimized training code for the mixture-of-experts architecture, and even the novel reinforcement learning method for training with the scientific community through open-source and open-access publications [8–11]. These resources should empower researchers in radiological clinics to train and deploy highly efficient mixture-of-experts models, as suggested by Sorin et al.
